# Genetic determinants of macrolide and tetracycline resistance in penicillin non-susceptible *Streptococcus pneumoniae* isolates from people living with HIV in Dar es Salaam, Tanzania

**DOI:** 10.1186/s12941-023-00565-3

**Published:** 2023-02-20

**Authors:** Joel Manyahi, Sabrina J. Moyo, Nina Langeland, Bjørn Blomberg

**Affiliations:** 1 Department of Clinical Science, University of Bergen, Bergen, Norway; 2 National Advisory Unit for Tropical Infectious Diseases, Department of Medicine, Haukeland University Hospital, Bergen, Norway; 3 Department of Microbiology and Immunology, Muhimbili University of Health and Allied Sciences, P.O. Box 65001, Dar es Salaam, Tanzania

## Abstract

**Background:**

Over one million yearly deaths are attributable to *Streptococcus pneumoniae* and people living with HIV are particularly vulnerable. Emerging penicillin non-susceptible *Streptococcus pneumoniae* (PNSP) challenges therapy of pneumococcal disease. The aim of this study was to determine the mechanisms of antibiotic resistance among PNSP isolates by next generation sequencing.

**Methods:**

We assessed 26 PNSP isolates obtained from the nasopharynx from 537 healthy human immunodeficiency virus (HIV) infected adults in Dar es Salaam, Tanzania, participating in the randomized clinical trial CoTrimResist (ClinicalTrials.gov identifier: NCT03087890, registered on 23rd March, 2017). Next generation whole genome sequencing on the Illumina platform was used to identify mechanisms of resistance to antibiotics among PNSP.

**Results:**

Fifty percent (13/26) of PNSP were resistant to erythromycin, of these 54% (7/13) and 46% (6/13) had MLS_B_ phenotype and M phenotype respectively. All erythromycin resistant PNSP carried macrolide resistance genes; six isolates had *mef*(A)-*msr*(D), five isolates had both *erm*(B) and *mef*(A)-*msr*(D) while two isolates carried *erm*(B) alone. Isolates harboring the *erm*(B) gene had increased MIC (> 256 µg/mL) towards macrolides, compared to isolates without *erm*(B) gene (MIC 4-12 µg/mL) p < 0.001. Using the European Committee on Antimicrobial Susceptibility Testing (EUCAST) guidelines, the prevalence of azithromycin resistance was overestimated compared to genetic correlates. Tetracycline resistance was detected in 13/26 (50%) of PNSP and all the 13 isolates harbored the *tet*(M) gene. All isolates carrying the *tet*(M) gene and 11/13 isolates with macrolide resistance genes were associated with the mobile genetic element Tn*6009* transposon family. Of 26 PNSP isolates, serotype 3 was the most common (6/26), and sequence type ST271 accounted for 15% (4/26). Serotypes 3 and 19 displayed high-level macrolide resistance and frequently carried both macrolide and tetracycline resistance genes.

**Conclusion:**

The *erm*(B) and *mef*(A)-*msr*(D) were common genes conferring resistance to MLS_B_ in PNSP. Resistance to tetracycline was conferred by the *tet*(M) gene. Resistance genes were associated with the Tn*6009* transposon.

## Background

*Streptococcus pneumoniae* is a transient colonizer of the nasopharynx, with colonization peaking in the early years of life and declining into adulthood. It is associated with both invasive and non-invasive pneumococcal diseases. The incidence of invasive pneumococcal diseases is most prominent at the extremes of age, as well as in immunocompromised hosts and people with chronic respiratory tract diseases. People living with HIV are particularly vulnerable to severe pneumococcal disease [1] Globally, *Streptococcus pneumoniae* is estimated to have caused as many as 515,000 deaths (95% uncertainty interval, UI 302,000–609,000) in children aged < 5 years in 2015, and approximately 50% of those deaths occurred in four countries in Africa (Nigeria, Democratic republic of Congo) and Asia (India, Pakistan) [2]. Globally, *Streptococcus pneumoniae* is estimated to cause 1,189,937 deaths (UI 690,445-1,770,660) [3].

Before 1970, pneumococci were readily susceptible to nearly all relevant antibiotics, and penicillin was the antibiotic of choice. In the late 1970s, pneumococci with non-susceptibility to penicillin emerged, resulting in treatment failures [4, 5]. The discovery of pneumococci resistant to penicillin shifted empirical treatment for suspected bacterial respiratory tract infection to macrolides and tetracycline. In Tanzania, standard treatment guidelines recommend macrolides and tetracyclines as first and second line treatments, respectively, for mild to moderate community acquired pneumonia caused by *Streptococcus pneumoniae* [6]. However, the recommendation is not based on current evidence of susceptibility patterns, as surveillance of the trend of antibiotic resistance in *Streptococcus pneumoniae* is limited in Tanzania. Data from the Network for Surveillance of Pneumococcal Disease in the East African Region in the pre-pneumococcal vaccination era reported a low rate of *Streptococcus pneumoniae* resistant to erythromycin and other antibiotics in Tanzania [7]. Furthermore, a meta-analysis of childhood pneumococcal diseases in Africa prior to the widespread use of the pneumococcal capsular vaccine (PCV) reported a low rate of resistance to erythromycin, but a substantially higher rate of resistance to tetracycline [8].

However, post-PCV surveillance studies conducted in well-organized settings have shown increased pneumococcal resistance to erythromycin and other antibiotics, partly attributed to increased consumption of macrolides [9–11].

Pneumococcal resistance to macrolides is mediated by erythromycin ribosomal methylase B (*erm*(B)) encoding enzymes that methylate the 23S rRNA, thereby inhibiting macrolide binding [12]. The *erm*(B) confers resistance to macrolides, lincosamides, and Streptogramin B, producing MLS_B_ phenotypes [13, 14]. Macrolide efflux protein A and E, efflux pumps encoded by the *mef*(A) and *mef*(E) genes, and ribosomal mutations (23S rRNA), are other common causes of macrolide resistance in *Streptococcus pneumoniae* [13]. The *mef*(A/E) genes confer the M phenotype, exhibiting low level resistance to macrolides, but not resistance to lincosamides and streptogramin B. The macrolide resistance genes are commonly carried on mobile genetic elements, facilitating their easy intra- and interspecies dissemination [15, 16]. The Tn*916* transposon family that contains the tetracycline resistance determinant *tet*(M), has frequently been reported to harbor macrolide resistance determinant genes [17].

Macrolide resistance determinants vary with geographical locations [13]. In Tanzania, where macrolides and tetracyclines are commonly used and easily accessible over the counter without prescriptions, the mechanisms of resistance to these antibiotics in *Streptococcus pneumoniae* has not been studied. Therefore, we performed this study using whole genome sequencing to determine mechanisms of antibiotic resistance among penicillin non-susceptible *Streptococcus pneumoniae* isolated from Tanzania.

## Materials and methods

### Bacterial isolates

Twenty-six penicillin non-susceptible *Streptococcus pneumoniae* were isolated by culturing nasopharyngeal swabs obtained from healthy HIV infected adults in Tanzania as part of the randomized clinical trial CoTrimResist (ClinicalTrials.gov identifier: NCT03087890, registered on 23rd March, 2017). The study population and bacterial isolates have been described previously [18]. *Streptococcus pneumoniae* was identified by conventional methods including optochin disk and bile susceptibility and further confirmation was done by Matrix-Assisted Laser Desorption/Ionization-Time of Flight (MALDI-TOF) mass spectrometry (MS), using the Microflex LT instrument and MALDI Biotyper 3.1 software (Bruker Daltonics, Bremen, Germany). Isolates with discordant results between MALDI-TOF and conventional identification with optochin disk and bile susceptibility were omitted.

Serotyping of *Streptococcus pneumoniae* was performed by latex agglutination (Immulex™ Pneumotest Kit; SSI Diagnostica A/S, Hillerød, Denmark).

### Antimicrobial susceptibility testing

The E-test strip (bioMérieux, Marcy-I-Etoile, France) was used to determine the minimum inhibitory concentrations (MIC) for azithromycin, erythromycin and penicillin. The disk diffusion method was used to determine tetracycline and clindamycin susceptibility [19]. Muller Hinton supplemented with 5% sheep blood agar was used for antimicrobial susceptibility testing, and it was incubated at 35 °C in 5% CO_2_ for 20–24 h. The guidelines of the Clinical and Laboratory Standards Institute [19], and The European Committee on Antimicrobial Susceptibility Testing [20], were used to interpret antimicrobial susceptibility testing results. PNSP was defined according to CLSI breakpoint interpretation [19].

### Whole genome sequencing and analysis

Whole genome sequencing was performed using the Next generation sequencing platform HiSeq X10 (Illumina, San Diego, CA, USA) at MicrobesNG (Microbes NG, Birmingham, UK). Quality filtering and sequencing short read trimming were performed by MicrobesNG using SPAdes and annotated in GenBank. Short read sequences were assembled using Unicycler at MicrobesNG.

For allocation of multi-locus sequence typing (MLST) and clonal complex, we used the online MLST database website https://pubmlst.org/.

Identification of acquired resistance was performed using the web-based platform ResFinder v3.2 of the Center for Genomic Epidemiology (http://www.genomicepidemiology.org/).

For identification of mobile genetic elements and their related acquired antimicrobial resistance we used the Center for Genomic Epidemiology MobileElement Finder v1.0.3 (http://www.genomicepidemiology.org/).

This Whole Genome Shotgun project has been deposited at DDBJ/ENA/GenBank under the BioProject number PRJNA918594.

### Statistical analysis

Categorical variables were presented in frequencies, percentages, and proportions. Categorical variables were compared using chi square test. A p-value < 0.05 was considered as threshold for statistical significance. Statistical analysis was performed using STATA version 16 (College Station, TX).

## Results

A total of 26 penicillin non-susceptible *Streptococcus pneumoniae* were analyzed by whole genome sequencing. Resistance to both macrolides and tetracyclines was observed in 12/26 (46%) of PNSP isolates.

Phenotypic results using both EUCAST and CLSI breakpoint interpretation showed that 13/26 (50%) of PNSP isolates were resistant to macrolides (erythromycin). Phenotypic resistance to erythromycin was in concordance with genotypic resistance determinants as shown in Table 1.

**Table 1:** Phenotypic and Genotypic characteristics of penicillin non-susceptible *Streptococcus pneumoniae*

Strain number	Isolation Year	Serotype	ST	PEN MIC	AZT MIC	ERY MIC	MLS_B_ genes			TET Disc diffusion	TET genes	Plasmid	Tn	CHL
2002c	2019	19F	5339	0.25	1.5	0.19	–	–		S	–	–	–	–
2145c	**2019**	34	5258	0.032	2	0.19	–	–	–	S	–	–		–
258d	**2019**	35B	6b3a	**0.125**	**96**	**8**	–	*mef*(A)	*Msr*(D)	R	*Tet*(M)	repUS43	6009	–
469c	**2018**	35B	172	**0.38**	**32**	**12**	–	*mef*(A)	*Msr*(D)	S	–	–	–	–
2010a	**2018**	19A	847	**0.50**	**256**	**256**	*erm*(B)		–	R	*Tet*(M)	repUS43	6009	-
263c	**2018**	6A	3460	0.125	1	0.125	–	–	–	S	–	–	–	–
57c	**2017**	3	271	**1.5**	**256**	**256**	*erm*(B)	*mef*(A)	*Msr*(D)	R	*Tet*(M)	repUS43	6009	–
2267a	**2018**	3	271	**0.25**	**256**	**256**	*erm*(B)	*mef*(A)	*Msr*(D)	R	*Tet*(M)	repUS43	6009	–
90d	**2018**	15A	0c86	0.25	1	0.094	–	–	–	S	–	–	–	
498d	**2018**	11A	14821	**0.25**	**6**	**4**		*mef*(A)	*Msr*(D)	R	*Tet*(M)	repUS43	6009	*Cat*(pC194)
116d	**2018**	11A	14821	**0.19**	**32**	**4**		*mef*(A)	*msr*(D)	R	*tet*(M)	repUS43	6009	*Cat*(pC194)
380a	**2017**	38	6103	0.25	1	0.094	–	–	–	S	–	–	–	–
2071a	**2018**	3	2054	0.25	0.5	0.064	–	–	–	S	–	rep36	–	–
2019b	**2018**	4	9a19	0.50	1	0.125	–	–	–	S	–	–	–	–
2267b	**2018**	3	271	**0.75**	**256**	**256**	*erm*(B)	*mef*(A)	*msr*(D)	R	*tet*(M)	repUS43	6009	–
2052d	**2019**	3	700	0.38	0.75	0.094	–	–	–	R	*tet*(M)	repUS43	6009	
219a	**2017**	35B	172	0.25	1	0.094	–	–	–	S	–	–	–	–
268a	**2017**	23B	6fe5	0.25	0.75	0.125	–	–	–	S	–	–	–	–
2034b	**2018**	3	271	**0.38**	**256**	**256**	*erm*(B)	*mef*(A)	*msr*(D)	R	*tet*(M)	repUS43	6009	
393a	**2017**	35B	172	0.25	1	0.094	–	–	–	S	–	–	–	–
2164a	**2018**	19F	cf17	**0.25**	**32**	**6**	–	*mef*(A)	*msr*(D)	R	*tet*(M)	rep13	6009	*Cat*(pC194)
272b	**2017**	19A	0c11	**0.25**	**256**	**256**	*erm*(B)	–	–	R	*tet*(M)	repUS43	6009	–
258b	**2017**	21	cdc8	0.047	1	0.125	–	–	–	S	–	–	–	–
252d	**2017**	46	15772	**0.016**	**24**	**8**	–	*mef*(A)	*msr*(D)	R	*tet*(M)	repUS43	6009	–
2014d	**2019**	19F	8678	**0.75**	**256**	**256**	*erm*(B)	*mef*(A)	*msr*(D)	R	*tet*(M)	repUS43	6009	–
369b	**2018**	23F	1188	0.25	2	0.125	–	–	–	S	–	–	–	

*erm*(B) Macrolide, Lincosamide and Streptogramin B resistance, *mef*(A) Macrolide resistance, *msr*(D) Macrolide resistance, *tet*(M) Tetracycline resistance, *cat*(pC194) Chloramphenicol resistance, *AZT* Azithromycin, *ERY *Erythromycin, *PEN *Penicillin, *TET* Tetracycline, *CHL *Chloramphenicol, *ST *sequence typing, *MIC *minimum inhibitory concentration

For azithromycin resistance, using EUCAST breakpoints (MIC > 0.5 µg/mL), all PNSP (26/26) were interpreted as resistant, but genetic markers conferring macrolide resistance could only be found in isolates with MIC ≥ 6 µg/mL (50%, 13/26). Using CLSI interpretation breakpoints (MIC ≥ 2 µg/mL), 58% (15/26) of PNSP were interpreted as resistant to azithromycin, while genotypic markers for macrolide resistance was found in 87% (13/15) of these isolates (Table 1).

Genes conferring resistance to the group of macrolides, lincosamides, and streptogramin B were observed in all the 13-erythromycin resistant PNSP isolates. The MLS_B_ phenotype (resistance to macrolides, lincosamides, and streptogramin B) accounted for 54% (7/13) while the M phenotype (resistance to macrolides, but not to lincosamides or streptogramin B) accounted for the remaining 46% (6/13).

The macrolide efflux gene *mef*(A)-*msr*(D) was observed in 6 isolates. Five isolates carried both the erythromycin ribosomal methylase gene *erm*(B) and *mef*(A)-*msr*(D), while *erm*(B) alone was detected in only two isolates. Isolates carrying the *erm*(B) gene had increased MIC for both erythromycin and azithromycin, > 256 µg/mL, compared to isolates lacking the *erm*(B) gene (MIC 4-12 µg/mL), p < 0.001.

The *mef*(A)-*msr*(D) gene predicted phenotypic resistance to macrolides, but at low MIC values (4–12 µg/mL for erythromycin) and (6–96 µg/mL for azithromycin). Carrying the *mef*(A)-*msr*(D) gene alone did not predict phenotypic resistance to lincosamides (clindamycin) and the six isolates carrying *mef*(A)-*msr*(D) were all phenotypically susceptible to clindamycin.

Disc diffusion results showed that 13/26 (50%) of PNSP isolates were resistant to tetracyclines. All the 13-tetracycline resistant PNSP isolates carried the *tet*(M) gene which confers resistance to tetracyclines. Three PNSP isolates harbored cat (pC194) which confers resistance to chloramphenicol.

The *Tn*6009-like element was detected in 13 PNSP. All tetracycline resistant PNSP were associated with a *Tn*6009 like element, while 12/13 of the erythromycin resistant PNSP had a *Tn*6009 like element. Twelve out of 13 tetracycline-resistant PNSP isolates were associated with plasmid replicon type repUS43.

Serotype 3 was the most common, followed by serotype19 and 35B. The majority of serotype 3 and 19 PNSP displayed high level macrolide resistance and carried *erm*(B) and *tet*(M) genes. MLST analysis identified seventeen different sequence types (ST), among which ST271 accounted for 15% (4/26), followed by ST172 (12%, 3/26) and ST14821 (8%, 2/26). The ST271 isolate belonged to serotype 3 and carried multiple resistance-determinant genes.

## Discussion

We observed a discrepancy in azithromycin susceptibility depending on whether using breakpoints from EUCAST or CLSI guidelines for interpretation. All 13 PNSP isolates harboring genetic determinants for macrolide resistance were correctly identified as resistant to erythromycin and azithromycin (all with MIC ≥ 4 µg/mL) regardless of which breakpoints were used (sensitivity 100%, 13/13). Using the EUCAST breakpoints appeared to overestimate azithromycin resistance, as all 13 erythromycin-susceptible PNSP without genetic determinants of macrolide resistance were interpreted as resistant to azithromycin (MIC-values from 0.5 to 2, specificity 0%, 0/13). Using CLSI breakpoints only misclassified two such isolates (MIC 2 µg/mL, specificity 85%, 11/13). Therefore, relying on current EUCAST guidelines appears to overestimate azithromycin resistance and could in the clinical perspective lead to unnecessary use of more broad-spectrum antibiotics. Our findings suggest that the EUCAST guidelines currently use a too low cutoff for MIC-values for azithromycin resistance in pneumococci.

PNSP susceptibility to erythromycin, on the other hand, was similar using both EUCAST and CLSI breakpoints, and the phenotypic findings correlated well with the identified genotypic resistance markers. To avoid variations in interpretation, our findings call for AST guidelines to be harmonized. Both CLSI and EUCAST state that erythromycin susceptibility can predict susceptibility to clarithromycin, azithromycin, dirithromycin, and roxithromycin [19, 20]. Because almost all PNSP resistant to erythromycin carried genetic determinants for macrolide resistance, our study supports that erythromycin determines susceptibility to other macrolides.

In Tanzania, macrolides are commonly used to treat respiratory tract infections. In the treatment of community-acquired pneumonia, erythromycin and azithromycin are used as first and second line treatment, respectively [6]. In this study, however, we observed that 50% and 58% of PNSP were resistant to erythromycin and azithromycin, respectively. Consequently, macrolides appear potentially ineffective for treating PNSP infections in this setting. This calls for prudent use of antibiotics including the use of narrow-spectrum penicillin. But for treatment failure or infections likely caused by resistant pneumococci/PNSP, options are difficult, with azithromycin, the currently preferred treatment covering just half of the PNSP.

The most common phenotype was MLS_B_ and isolates with this phenotype harbored the *erm*(B) gene conferring a high level of resistance to macrolides (> 256 µg/mL) and clindamycin. All but two isolates with the MLS_B_ phenotype carried the *mef*(A) and *msr*(D) genes as well. The *erm*(B) gene has been reported as the most common macrolide resistance determinant in *Streptococcus pneumoniae* in studies from Africa [14, 21, 22] and some part of Asia [11]. Macrolide resistance genes in *Streptococcus pneumoniae* have marked geographical variability [13]. The *mef*(A) has been reported to be the predominant mechanism of pneumococcal macrolide resistance in North America and some parts of Europe [13]. Our study found the macrolide efflux genes *mef*(A)/*msr*(D) to be more prevalent (11/13) than the *erm*(B) genes (7/13). Still, considering the high number of isolates harboring both types of resistance genes (5/13), the dominant MLS_B_ phenotype was more frequent (7/13, *erm*(B) with or without *mef*(A)/*msr*(D)) than the M-phenotype (6/13, *mef*(A)/*msr*(D) alone).

Previous studies have shown that tetracycline and macrolide resistance genes are carried on mobile genetic elements, composite conjugative transposons, Tn*916*-like elements, which facilitate their dissemination between different bacteria [23, 24]. Tn*916* and Tn*917*-like composite elements have been documented to facilitate dissemination of *erm*(B) and *mef*(A/E) in *Streptococcus pneumoniae* [17]. However, our study found a Tn*6009*-like element in all 13 PNSP isolates carrying the *tet*(M) gene which confers resistance to tetracycline, and in 12/13 (92%) of PNSP isolates carrying macrolide resistance determinants. Tn*6009* is a member of the Tn*916*–Tn*1545* transposon family previously detected in Gram-positive and Gram-negative bacteria [25]. Tn*6009* has been reported to carry genes conferring resistance against tetracycline *tet*(M), and inorganic and organic mercury [25]. Through horizontal gene transfer, the conjugative mobile elements enable bacteria to acquire and disseminate DNA between related and unrelated bacteria. The presence of transposons containing macrolide and tetracycline resistance genes in PNSP in our study could indicate an increased risk of dissemination of these resistance determinants.

## Conclusion

Macrolides and tetracyclines have only about 50% chance of being effective against PNSPs recovered from nasopharynx from people living with HIV in Dar es Salaam. The *erm*(B) and *mef*(A)-*msr*(D) were common genes conferring resistance to macrolides and clindamycin, while resistance to tetracycline was conferred by the *tet*(M) gene. Detection of the composite conjugate transposon Tn6009 associated with macrolides and tetracycline genes could indicate the possibility of horizontal transfer of resistant genes. Using EUCAST guidelines for interpretation overestimates azithromycin resistance in PNSP compared to genetic correlates of resistance.

## Data Availability

Data are available on reasonable request.
